# Supplementary material for: Identification of Freezing-Responsive microRNAs and Their Targets in Chinese Jujube by Small RNA and Degradome Sequencing
Source: Int J Mol Sci. 2026 Sep 4;27(17):7912. doi: 10.3390/ijms27177912 (PMC13566890; doi:10.3390/ijms27177912)
Supplement: Supplementary file 1 [file ijms-27-07912-s001.zip › Supplementary Material S2/novel_miR_3.pdf]

Provisional ID	:	novel_miR_3
Score total	:	0.9
Score for star read(s)	:	-1.3
Score for read counts	:	0
Score for mfe	:	0.6
Score for randfold	:	1.6
Score for cons. seed	:	
Total read count	:	264
Mature read count	:	221
Loop read count	:	43
Star read count	:	0

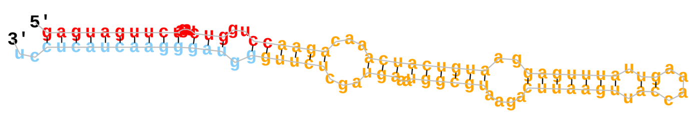

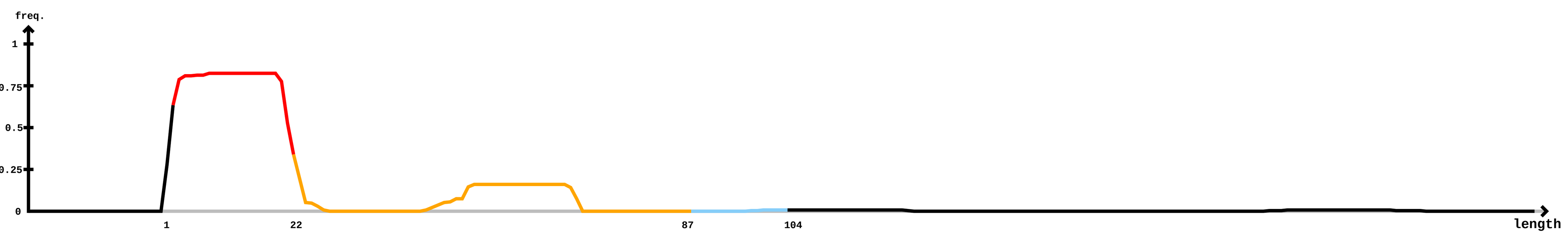

Mature Star

5'- uuauggcuacauaaaccauucagaguaguucuagaucugguccaagacaaacuacuguaaggaguuuauugaaaccauugaauucagaaugcgguaaaguagcucuuggguagggaacuacuccuuugauggguaucgcaauggcccuuuuugcgguuccuaucuauuguuuuauagauuuauaauucuuccauuuuacuagauggaauuggaaugaauuagauucacaaaacuaguaacuagcuuuuc -3' exp

....(((((............((((((((((....(((..((((((...(((((((((..(((((((.((....)).)))))))....))))))..)))...)))))).)))))))))))))....((((((.(((((((.........))))))).))))))(((((((((...((....((((((((((.(((........))).))))).))))))...))...))))).)))).)))))....

read	reads	mm	sample
agaguaguucuagaucuggu	18	0	LCK-1
agaguaguucuCgaucuggu	1	1	LCK-1
Cgaguaguucuagaucuggu	1	1	LCK-2
agaguaguucuagaNcuggu	1	1	LCK-1
agaguaguucuagaucuggu	2	0	LT-1
agaguaguucuagaucuggu	23	0	LCK-2
agaguaguucuagaucugguc	5	0	LT-2
agaguaguucuagaucugguc	19	0	JT-1
agaguaguucuagaucugguc	1	0	JCK-2
agaguaguucuagaucuggucca	1	0	LT-1
agaguaguucuagaucugguccaag	1	0	JCK-1
agaguaguucuagaucugguccaaga	2	0	LT-3
gaguaguucuagaucugg	11	0	JT-2
gaguaguucuagaucugg	1	0	JCK-3
gaguaguucuagaucugg	1	0	JT-3
gaguaguucuagaucuggu	1	0	LT-2
gaguaguucuagaucugguc	2	0	LT-2
gaguaguucuagaucugguc	2	0	LT-3
gaguaguucuagaucuggucc	30	0	LCK-1
gaguaguucuagaucuggucc	1	0	LT-1
gaguaguucuagauNuggucc	2	1	LCK-1
gaguaguucuagaucuggucc	6	0	LCK-3
gaguaguucuagaucuggucc	3	0	LT-2
gaguaguucuagaucuggucc	1	0	JCK-2
gaguaguucuagaucuggucc	28	0	LCK-2
gaguaguucuagaucuggucc	1	0	JCK-1
gaguaguucuagaucuggucc	2	0	LT-3
gaguaguucuagaucugguccaa	1	0	JCK-3
gCguaguucuagaucugguccaag	1	1	LT-2
gaguaguucuagaucugguccaag	1	0	LT-2
aguaguucuagaucuggu	8	0	JT-3
aguaguucuagaucuggu	2	0	LT-3
aguaguucuagaucuggu	1	0	JCK-1
aguaguucuagaucuggu	9	0	JT-2

Mature / **Star**

uuauggcuacauaaaccauuca**gaguaguucuagaucuggucc**aagacaaacuacuguaaggaguuuauugaaaccauugaauucagaaugcgguaaaguagcucuug**gguagggaacuacuccu**uugauggguaucgcaauggcccuuuuugcgguuccuaucuauuguuuuauagauuuauaauucuuccauuuuacuagauggaauuggaaugaauuagauucacaaaaacuaguaacuagcuuuuc

aguaguucuagaucugguc	6	0	JT-2
aguaguucuagaucugguc	2	0	JCK-2
aguaguucuagaucugguc	12	0	JT-3
aguaguucuagaucuggucca	1	0	LT-3
guaguucuagaucugguc	1	0	LT-3
guaguucuagaucuggucc	1	0	LT-1
guaguucuagaucugguccaaga	1	0	JCK-3
guaguucuagaucugguccaaga	3	0	JCK-1
aguucuagaucugguccaag	1	0	LT-3
uucuagaucugguccaag	1	0	LT-1
uucuagaucugguccaagac	2	0	LT-3
uuuauugaaaccauugaauucagaau	2	0	LCK-3
uuauugaaaccauugaauucagaau	1	0	LT-1
uuauugaaaccauugaauucagaau	1	0	JT-2
uuauugaaaccauugaauucagaau	2	0	JT-3
uauugaaaccauugaauucagaau	1	0	JT-2
uauugaaaccauugaauucagaau	3	0	JT-3
auugaaaccauugaauucagaa	4	0	LT-2
uugaaaccauugaauucagaa	1	0	LT-1
ugaaaccauugaauucaga	2	0	JT-2
ugaaaccauugaauucaga	3	0	JT-3
aaaccauugaauucagaa	6	0	JT-2
aaaccauugaauucagaa	7	0	JT-3
aaaccauugaauucagaau	1	0	LT-1
aaaccauugaauucagaau	5	0	LT-3
aaccauugaauucagaau	1	0	JT-2
aaccauugaauucagaau	2	0	JT-3
aaccauugaauucagaau	1	0	LT-2
uacuccuuugauggguaucgcaauggc	1	0	LT-2
cuccuuugauggguaucgcaaugg	1	0	JT-3
ggaauuggaaugaauuagauucacaC	1	1	LT-3
auuggaaugaauuagauu	1	0	LT-1
